# Beta-blockers after myocardial infarction: time to change the guidelines

**DOI:** 10.1093/ehjcvp/pvag001

**Published:** 2026-02-03

**Authors:** Tomas Jernberg

**Affiliations:** Department of clinical sciences, Danderyd Hospital, Karolinska Institutet, Stockholm, Sweden

The role of long-term beta-blocker therapy in patients with myocardial infarction (MI) and preserved ejection fraction (EF) receiving contemporary care has increasingly been questioned. Over the past two years, randomized trials and individual patient-level meta-analyses have substantially clarified the evidence base.^1-5^

The REDUCE-AMI trial, presented in 2024, enrolled patients with MI and preserved EF (≥50%) who underwent coronary angiography with revascularization when clinically indicated.^1^ Beta-blocker therapy did not reduce the risk of the primary endpoint of death or MI. Although the trial was powered to detect a 25% relative risk reduction, the overlapping time-to-event curves and consistent findings across all pre-specified subgroups and secondary endpoints argued strongly against a clinically meaningful benefit. Despite these results, which were acknowledged in the 2025 ACC/AHA/ACEP/NAEMSP/SCAI acute coronary syndrome guidelines, recommendations remained unchanged.

Additional evidence has recently emerged. In a pooled analysis of the Danish DANBLOCK and Norwegian BETAMI trials, beta-blocker therapy was associated with a 15% reduction of a broad composite endpoint including all-cause death, MI, unplanned revascularization, ischemic stroke, heart failure, or malignant ventricular arrhythmia among patients with preserved (≥50%) or mildly reduced (40%–49%) EF.^3^ Importantly, the benefit appeared larger in patients with mildly reduced EF. In contrast, the larger Spanish–Italian REBOOT trial showed no benefit of beta-blockers in patients with preserved or mildly reduced EF for all-cause death, reinfarction, or hospitalization for heart failure.^5^

Subsequently, two separate pre-specified individual patient-level meta-analyses have addressed the two different EF strata. In the first, pooling data from DANBLOCK/BETAMI, REBOOT and CAPITAL-RCT among patients with mildly reduced EF (40%–49%), beta-blocker therapy reduced the risk of death, reinfarction, or hospitalization for heart failure.^2^ In contrast, the second meta-analysis restricted to patients with preserved EF (≥50%), combining data from REDUCE-AMI, DANBLOCK/BETAMI, REBOOT, and CAPITAL-RCT, found no evidence of benefit for any endpoint or subgroup.^4^ Taken together, these findings support continued beta-blocker use in patients with MI and EF <50%, but not routine use in those with preserved EF.

Concerns have been raised regarding the pragmatic, open-label design of these trials and lower adherence to assigned treatment compared with traditional randomized controlled trials.

However, the primary endpoints are unlikely to have been materially influenced by lack of blinding, and per-protocol analyses were consistent with intention-to-treat results. Other criticisms have focused on patient selection and the predominant use of beta-1-selective blockers. In REDUCE-AMI, nearly half of eligible patients were enrolled, and the evidence supporting beta-1-selective blockers in cardiovascular diseases is substantial, including the meta-analysis in patients with mildly reduced EF.^2^

Importantly, these trials excluded patients with indications for beta-blockers beyond secondary prevention. Patients with MI and reduced EF (<50%) should continue to receive beta-blockers, and patients with tachyarrhythmias, angina, or hypertension not controlled by first-lite treatment may also benefit. However, for patients with MI, preserved EF (≥50%), and no other indication, the guidelines should be changed, as routine long-term beta-blocker therapy offers no proven benefit while exposing patients to potential adverse effects.

## Data Availability

This article does not contain any original data.
